# Multiple quantum filtered ^23^Na NMR in the Langendorff perfused mouse heart: Ratio of triple/double quantum filtered signals correlates with [Na]_i_

**DOI:** 10.1016/j.yjmcc.2015.07.009

**Published:** 2015-09

**Authors:** Thomas R. Eykyn, Dunja Aksentijević, Karen L. Aughton, Richard Southworth, William Fuller, Michael J. Shattock

**Affiliations:** a Department of Imaging Chemistry and Biology, Division of Imaging Sciences and Biomedical Engineering, King's College London, King's Health Partners, St. Thomas' Hospital, London SE1 7EH, United Kingdom; b The British Heart Foundation Centre of Research Excellence, The Rayne Institute, King's College London, St. Thomas' Hospital, London SE1 7EH, United Kingdom; c Division of Cardiovascular and Diabetes Medicine, University of Dundee, Dundee, United Kingdom

**Keywords:** Multiple quantum filtered ^23^Na, TQF, DQF, Langendorff perfused mouse heart, Shift reagent

## Abstract

We investigate the potential of multiple quantum filtered (MQF) ^23^Na NMR to probe intracellular [Na]_i_ in the Langendorff perfused mouse heart. In the presence of Tm(DOTP) shift reagent the triple quantum filtered (TQF) signal originated largely from the intracellular sodium pool with a 32 ± 6% contribution of the total TQF signal arising from extracellular sodium, whilst the rank 2 double-quantum filtered signal (DQF), acquired with a 54.7° flip-angle pulse, originated exclusively from the extracellular sodium pool. Given the different cellular origins of the ^23^Na MQF signals we propose that the TQF/DQF ratio can be used as a semi-quantitative measure of [Na]_i_ in the mouse heart. We demonstrate a good correlation of this ratio with [Na]_i_ measured with shift reagent at baseline and under conditions of elevated [Na]_i_. We compare the measurements of [Na]_i_ using both shift reagent and TQF/DQF ratio in a cohort of wild type mouse hearts and in a transgenic PLM^3SA^ mouse expressing a non-phosphorylatable form of phospholemman, showing a modest but measurable elevation of baseline [Na]_i_. MQF filtered ^23^Na NMR is a potentially useful tool for studying normal and pathophysiological changes in [Na]_i_, particularly in transgenic mouse models with altered Na regulation.

## Highlights

• Intracellular Na concentration [Na]_i_ is a key modulator of cardiac cell function.
• We developed an NMR-compatible Langendorff mouse heart perfusion system.
• The ratio of triple/double quantum filtered ^23^Na NMR signals correlates with [Na]_i_.
• Intracellular [Na]_i_ can be quantified under physiological perfusion conditions.
• The PLM^3SA^ transgenic mouse model has a measurable elevation of [Na]_i_ at baseline.

## 1 Introduction

Intracellular Na concentration [Na]_i_ is a key modulator of cardiac cell function [1]. At steady-state [Na]_i_ has been measured between 4–16 mM depending on species and preparation [2]. The Na electrochemical gradient across the cell membrane provides the energy for the action potential upstroke, as well as the active transport of many other ions, amino acids and substrates into the cell [2,3]. [Na]_i_ is also critical for the control of intracellular calcium [Ca]_i_ via the sodium–calcium exchanger (NCX), thereby determining sarcoplasmic reticulum (SR) Ca content and cardiac contractility [4]. Maintenance of the Na^+^ gradient is therefore fundamentally important in normal physiology but is also critically important in cardiac hypertrophy and heart failure where the elevation of [Na]_i_ contributes to contractile and electrical dysfunction [5–7].

Historically, many techniques have been used to measure intracellular Na but few are physiologically relevant, either measuring Na at room temperature or in quiescent preparations [1]. ^23^Na nuclear magnetic resonance (NMR) is able to distinguish intra versus extracellular Na pools, employing paramagnetic shift reagents such as Tm(DOTP) [8] to separate the two. However, these reagents are efficient chelators of Ca and Mg leading to modified ion homeostasis and reduced cardiac contractility [9]. As a result, shift reagents exhibit significant toxicity precluding their use in vivo. In contrast, multiple quantum filtered (MQF) ^23^Na nuclear magnetic resonance has shown great potential to probe intra and extracellular pools of Na in the *absence* of shift reagent and therefore under more physiological conditions [10,11]. Much work has been carried out developing multiple quantum filtered ^23^Na NMR in the perfused rat heart [10,12,13], in vivo in skeletal muscle and the brain [14] and in tumours [15]. In the perfused rat heart, the ^23^Na TQF signal has contributions from the intra and extracellular Na pools [11,12,16] and has been shown to be proportional to intracellular [Na]_i_ measured by atomic absorption spectroscopy [13]. Methods have been developed to suppress the extracellular contribution to the TQF signal [17]. On the other hand, the ^23^Na DQF signal was found previously to arise only from the extracellular Na pool in the rat heart suggesting that TQF and DQF ^23^Na NMR signals arise from different cellular compartments [18].

Whilst the isolated rat heart has historically been an important model for cardiovascular research, the ability to rapidly and efficiently manipulate the genome of the mouse has recently made this the species of choice for many physiological and biochemical studies. The application of MQF ^23^Na NMR techniques to the isolated perfused mouse heart is much more challenging due to technical issues of the smaller heart size and, prior to this study, has not been described. The aim of the present study was therefore to revisit the utility of the multiple quantum filtered experiment and to establish and characterise the methods necessary to apply MQF ^23^Na NMR measurements to isolated Langendorff-perfused mouse hearts in both wild-type and a transgenic PLM^3SA^ genotype displaying altered [Na]_i_ homeostasis. The PLM^3SA^ transgenic mouse has three mutations of the PLM phosphorylation sites Ser63, 68 and 69 which have been substituted for alanine, rendering PLM unphosphorylatable, leading to altered Na/K ATPase activity and elevated intracellular Na [6].

## 2 Methods

### 2.1 Langendorff mouse heart perfusion

C57BL/6J male mice (~ 28 g body weight) were purchased from Harlan (Harlan, UK). PLM^3SA^ and WT mice were bred ‘in house’ as previously described [6]. Mice were kept in individually ventilated cages with a 12 h light–dark cycle, controlled humidity and temperature (20–22 °C), fed standard chow and water ad libitum. All experiments were approved by institutional ethical review committee and conform to the UK Home Office Guidance on the Operation of the Animals Scientific Procedures Act 1986 (HMSO).

Mice were administered terminal anaesthesia via intra-peritoneal pentobarbitone injection (~ 140 mg/kg body weight). Hearts were rapidly excised, cannulated and perfused in isovolumic Langendorff mode and perfused at 80 mm Hg pressure maintained by a STH peristaltic pump controller feedback system (AD Instruments, Oxford, UK), at 37 °C with Krebs–Henseleit (KH) buffer continuously gassed with 95% O_2_/5% CO_2_ (pH 7.4) containing (in mM): NaCl 116, KCl 4.7, MgSO_4_·7H_2_O 1.2, NaHCO_3_ 25, KH_2_PO_4_ 1.4, CaCl_2_ 1.4, glucose 11 [19]. Cardiac function was assessed using a fluid-filled cling-film balloon inserted into left ventricle (LV), and connected via a rigid polyethylene line to an MR-compatible pressure transducer (DTX Plus TNF-R, Becton-Dickinson) and a PowerLab system (AD Instruments, Australia). The volume in the intraventricular balloon was adjusted using a 1.0 ml syringe to achieve an initial LV diastolic pressure of ~ 9 mm Hg. Left ventricular developed pressure (LVDP) was calculated from the difference between systolic (SP) and diastolic pressures (DP). Functional parameters (systolic pressure, end-diastolic pressure, heart rate, LVDP, coronary flow, perfusion pressure) were monitored using LabChart software v.7 (AD Instruments, Australia) throughout the experiment [19].

**Fig. 1:**
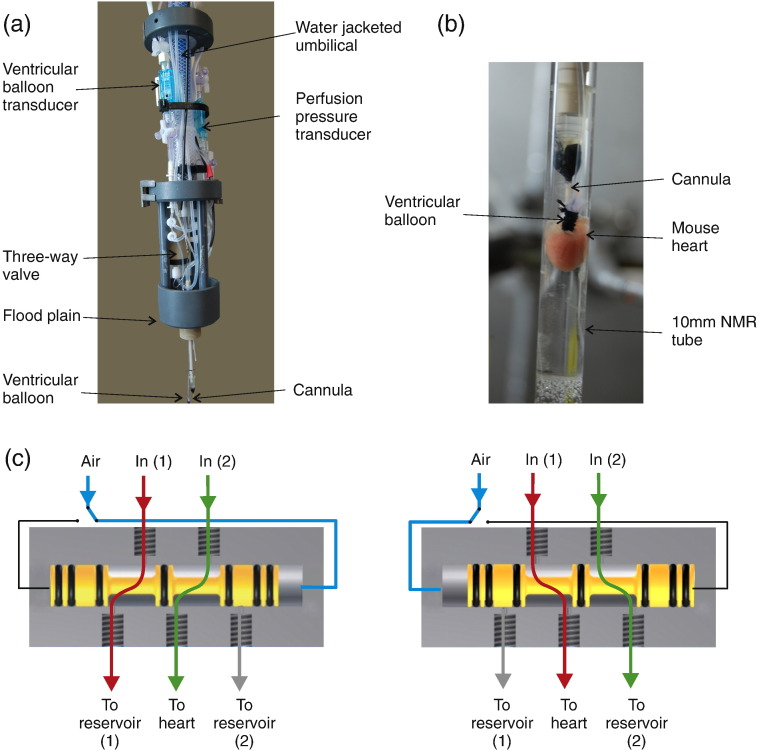
NMR compatible Langendorff constant pressure perfusion system (a, b) and a schematic of the pneumatically driven three-way valve to allow switching between reservoirs with minimum dead-volume (c).

The low flow rates and the long perfusion lines in NMR systems can create problems with adequate temperature control. In addition, these long perfusion lines create significant ‘dead-spaces’ (where temperature, gas solubility and pH may change) and introduce long delays on switching solutions. These problems were solved in the current system by the use of two parallel continuously recirculating water-jacketed perfusion lines (Fig. 1a). Control, and test solutions were gassed at 37 °C before being pumped (Gilson Minipuls 3) through two parallel lines within a water-jacketed (37 °C) umbilical line (approx 3 m in length). These two lines then enter a novel three-way pneumatically operated valve positioned directly above the cannula (Fig. 1a). Control solution was passed via the pneumatic valve to the heart whilst the test solution was recirculated back up the umbilicus to the external reservoir. When the valve is activated, the control solution is recirculated and the test solution passes to the heart with effectively no distal stagnant dead-space and a switching time of < 2 s, at the level of the heart. In order to protect the magnet, coils etc. from accidental leakage, the perfusion system includes a ‘flood plain’ between the perfusion lines, valve etc. and the cannula (Fig. 1a). This flood plain includes a moisture sensor connected to a remote alarm. The umbilical line, transducers, valve and all fittings were manufactured exclusively of non-magnetic parts and was fed via the top of the magnet and lowered into the active region of the bore/microimaging coil. At the end of each experiment, the perfusion system was removed from the magnet and hearts were immediately snap frozen using Wollenberger tongs, pre-cooled in liquid nitrogen, and wet and dry weights were recorded.

### 2.2 NMR spectroscopy

All experiments were carried out on a Bruker Avance III 400 MHz wide-bore spectrometer (Bruker, Karlsruhe, Germany) equipped with triple-axis gradients, a microimaging probe and exchangeable RF coil inserts (10 mm ^23^Na coil or ^1^H/^31^P dual tune coil) that enable rapid switching between different nuclei. NMR tubes (O.D. 10 mm) (Wilmad, UK) were shortened in length to just greater than the NMR active coil region in order to minimize the perfusate dead volume beneath the heart. The bore of the NMR was maintained (~ 313 K) by continuous delivery of warm water, from a thermostatically controlled water bath, through the imaging gradients of the MR. Temperature calibration was performed in situ using a capillary containing ethylene glycol at the position of the heart.

To analyse the relationship between [Na]_i_ elevation and TQF and DQF signal intensities, hearts were equilibrated for 20 min, followed by 20 min treatment with: (i) standard KHB (n = 6) (ii) 50 μM ouabain (n = 6), (iii) K^+^-free buffer (n = 3), (iv) K^+^-free/Ca^2 +^-free (n = 3) or (v) K^+^-free/Ca^2 +^-free/Mg^2 +^-free (n = 3) buffer. Interleaved TQF and DQF NMR acquisitions were recorded throughout the stability period and during the intracellular sodium elevation protocols. At the end of each intervention, perfusate was switched to KHB containing 5 mM Tm(DOTP)^5 −^ to shift the extracellular [Na]_e_ signal and quantify [Na]_i_. An intracellular volume of 2.5 ml/g dry weight of tissue was assumed for calculation of [Na]_i_ [20].

In order to confirm that cardiac energetics were normal and not compromised prior to any elevation in [Na]_i_, fully relaxed ^31^P experiments were also performed with the same perfusion system and phosphate free Krebs–Henseleit buffer, acquired with a 60° flip angle, 256 scans, a repetition time of 3.8 s and a total experiment duration of 16 min.

### 2.3 Multiple quantum filtered ^23^Na MRS

The multiple quantum filtered pulse sequence consists of [10]: 90°(ϕ_1_) − τ_m_ / 2 − 180°(ϕ_2_) − τ_m_ / 2 − β°(ϕ_2_) − δ − β°(ϕ_3_) − FID(ϕ_rec_). DQF experiments were acquired with a four-step phase cycle: ϕ_1_ = ϕ_2_ = 90°, 180°, 270°, 0°; ϕ_3_ = 0°; ϕ_rec_ = 0°, 180°, 0°, 180°. The flip angle β was set either to the magic angle, 54.7° (rank 2 T_2,±2_ only) or to 90° (both rank 2 T_2,±2_ and rank 3 T_3,±2_). TQF experiments were acquired with a six-step phase cycle: ϕ_1_ = 30°, 90°, 150°, 210°, 270°, 330°; ϕ_2_ = 120°, 180°, 240°, 300°, 0°, 60°; ϕ_3_ = 0°; ϕ_rec_ = 0°, 180°, 0°, 180°, 0°, 180° with the flip angle β = 90° (rank 3 T_3,±3_ only). The mixing time (τ_m_ = 3.6 ms) was calibrated for the maximum TQF signal in the mouse heart and set to be the same for the DQF experiments. All MQF experiments were acquired with 192 scans, 2048 data points, sweep width of 50 ppm, an acquisition time of 200 ms, pre-scan delay of 200 ms and a total acquisition time of 1.24 min. An exponential line broadening factor of 10 Hz was applied prior to Fourier transformation and subsequent baseline correction.

### 2.4 Shift reagent ^23^Na MRS

**Fig. 2:**
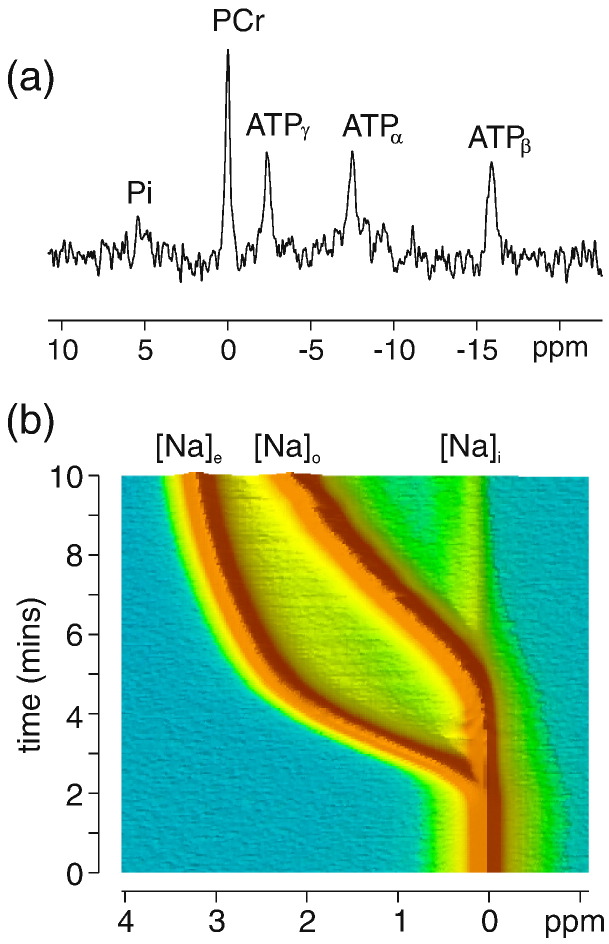
(a) Example ^31^P spectrum acquired during baseline stability period showing normal cardiac energetics. (b) Time-series of ^23^Na NMR spectra acquired in a perfused mouse heart during the infusion of Tm(DOTP) shift reagent. An initial shift of the intravascular extracellular signal [Na]_e_ is observed, followed by the signal from the buffer surrounding the heart [Na]_o_ was achieved within a 5–10 min timeframe.

All hearts were perfused at the end of the protocol with modified KHB containing 5 mM Tm(DOTP)^5 −^ (thulium (III) 1,4,7,10-tetraazacyclododecane-N,N′N″,N‴tetra(methyl-enephosphonate)) (Macrocyclics, Dallas, USA) gassed with 95% O_2_/5% CO_2_ (pH 7.4) containing (in mM): NaCl 116, KCl 4.7, MgSO_4_.7H_2_O 1.2, NaHCO_3_ 25, KH_2_PO_4_ 1.4, CaCl_2_ 1.4, glucose 11. Due to the Ca chelating effect of Tm(DOTP)^5 −^, [Ca]_free_ was measured by blood gas analyser which was below detection limit, corresponding to almost complete chelation of free Ca by the DOTP ligand. ^23^Na acquisitions were recorded in real-time during the perfusion with the shift reagent until the extracellular [Na]_e_ peak was completely shifted (5–10 min) (Fig. 2b). Following infusion of shift reagent a single quantum spectrum was acquired with 128 scans, 2048 data points, sweep width of 50 ppm, an acquisition time of 200 ms, pre-scan delay of 200 ms and a total acquisition time of 50 s. An exponential line broadening factor of 10 Hz was applied prior to Fourier transformation and subsequent baseline correction. The resulting [Na]_i_ peak was referenced to a glass bulb containing 2.9 μmol NaCl with 10 mM Tm(DOTP)^5 −^ at the position of the heart. The Na present in the calibration bulb was assumed to be 100% NMR visible ie *f_o_* = 1.0. The visibility of intracellular Na is more controversial but, in these studies, was assumed to be *f_i_* = 0.4 and this was assumed to remain constant throughout the experimental protocol. [21–23].

### 2.5 Statistical analysis

Data were analysed and expressed as mean ± standard error of the mean (SEM), n = number of independent experiments. Where appropriate, statistical analysis was performed using Students t-test and relationship between variables analysed using Pearson correlation. P < 0.05 was considered statistically significant.

## 3 Results

Table 1 shows functional data at 5 min intervals during the 20 min stability period. Averaged over the 20 min period (± SEM), systolic pressure was 107 ± 6 mm Hg, end diastolic pressure (EDP) was 11 ± 2 mm Hg, left ventricular developed pressure (LVDP) was 96 ± 15 mm Hg, heart rate (HR) was 455 ± 66 bpm and coronary flow was 2.3 ± 0.2 ml/min.

Fig. 2(a) shows a representative ^31^P spectrum acquired in the mouse heart at baseline prior to intervention and is representative of normal cardiac energetics displaying resonances from adenosine triphosphate (ATP), phosphocreatine (PCr) and inorganic phosphate (Pi). Fig. 2(b) shows a time-course of ^23^Na spectra acquired during perfusion with Tm(DOTP)^5 −^. In the absence of shift reagent (time = 0) the total Na peak corresponds to the sum of contributions from intracellular [Na]_i_, extracellular/intravascular [Na]_e_, and from buffer surrounding the heart [Na]_o_. During infusion of shift reagent the ^23^Na signal from the extracellular/intravascular [Na]_e_ pool is shifted first and at later times (5–10 min) mixing of buffer surrounding the heart is achieved.

**Fig. 3:**
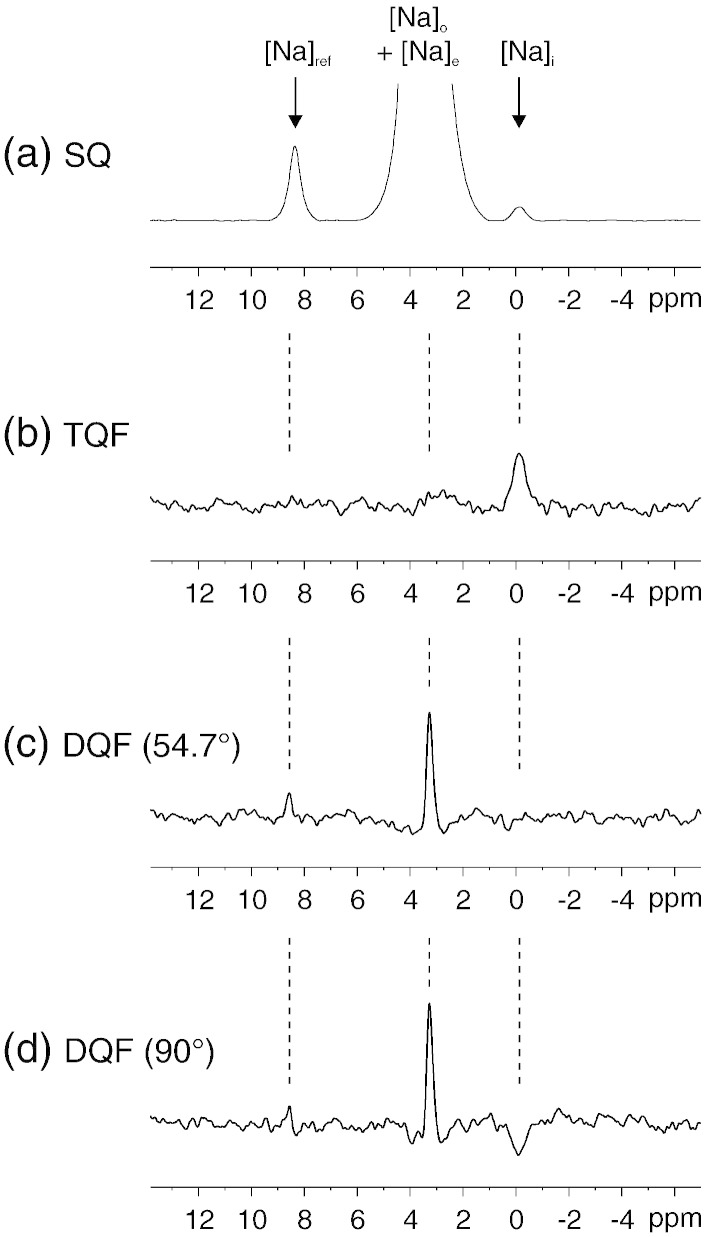
(a) ^23^Na single quantum spectrum post-infusion of shift reagent where the intracellular peak from [Na]_i_ is clearly discerned. (b) TQF spectrum acquired from the same heart as in (a) in the presence of shift reagent showing predominantly intracellular [Na]_i_ with a smaller contribution from extracellular [Na]_e_. (c) ^23^Na DQF spectrum acquired with a flip angle β = 54.7° showing predominantly extracellular [Na]_e_. (d) ^23^Na DQF spectrum acquired with a flip angle β = 90° showing both intracellular and extracellular Na.

Fig. 3(a) shows the single quantum ^23^Na NMR spectrum acquired after infusion of shift reagent where an unshifted Na signal arising from the intracellular [Na]_i_ pool, can readily be discerned and quantified. Fig. 3(b–d) shows representative ^23^Na MQF spectra acquired in the presence of shift reagent where the positions of the relevant peaks are denoted by dashed lines. Fig. 3(b) shows the TQF signal arising principally from the intracellular [Na]_i_ pool with a smaller contribution from the extracellular pool. The contribution to the total TQF signal arising from Na_e_ acquired with shift reagent was Na_e_/(Na_e_ + Na_i_) = 0.32 ± 0.06 (S.E. n = 6) corresponding to 32% of the total TQF signal. That is, in these experiments, 68% of the TQF signal reflects intracellular Na whilst 32% of the signal comes from extracellular sources. Fig. 3(c) shows the DQF spectrum acquired with shift reagent and a flip angle β = 54.7° showing exclusively extracellular Na_e_. Intracellular sodium was not observed employing the DQF sequence with a magic flip angle pulse. Fig. 3(d) shows the DQF spectrum acquired with shift reagent and a flip angle β = 90° showing both intracellular and extracellular Na and was not used further in this work due to a mixed contribution from both intra and extracellular Na pools.

**Fig. 4:**
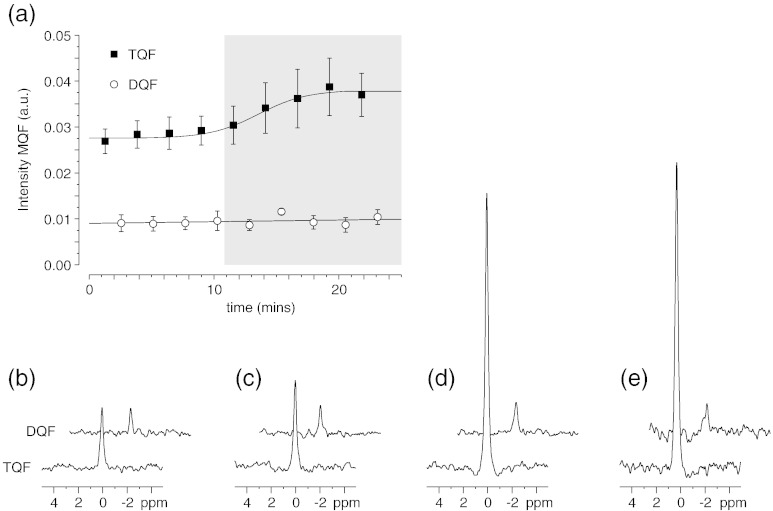
(a) Peak areas shown for a time-series (± S.E. n = 6) of ^23^Na TQF and DQF spectra acquired in an interleaved fashion in the perfused mouse heart during stability and during subsequent infusion of 50 μM ouabain, shaded area. Representative TQF and DQF spectra are shown; (b) during stability, (c) after 20 min infusion of ouabain, (d) after 20 min K^+^-free buffer and (e) after 20 min K^+^-free/Ca^2 +^-free buffer.

Fig. 4(a) shows the amplitude of the TQF and DQF signals acquired in an interleaved fashion during 20 min stabilisation period and during 20 min infusion of 50 μM ouabain. Representative ^23^Na TQF and DQF spectra are shown in Fig. 4(b) during baseline stability, (c) after 20 min infusion of ouabain (d) after 20 min K^+^-free buffer and (e) after 20 min K^+^-free/Ca^2 +^-free buffer. A significant increase in the TQF signal was observed with respect to the baseline TQF signal with minimal changes in the DQF signal. The increase in [Na]_i_ is consistent with the mode of action of ouabain and blocking of the Na/K ATPase or inhibition of Na efflux using modified buffers.

**Fig. 5:**
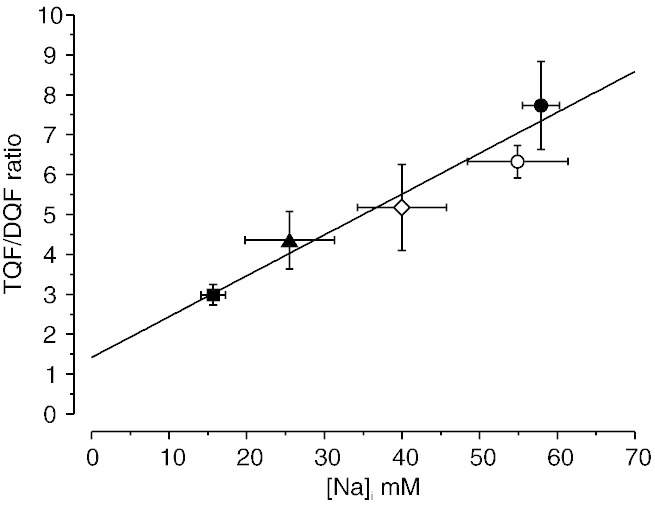
Plot of the TQF/DQF ratio R^TQF/DQF^ vs the measured concentration [Na]_i_ in the perfused mouse heart at baseline (filled square, n = 6) and following a 20 min intervention to elevate [Na]_i_; 50 μM ouabain (filled triangle, n = 6); 0 mM K^+^ (open diamond, n = 3), 0 mM K^+^/0 mM Ca^2 +^ (filled circle, n = 3) and 0 mM K^+^/0 mM Ca^2 +^/0 mM Mg^2 +^ (open circle, n = 3). Mean data are shown for each group ± S.E.

Fig. 5 shows a plot of the ratio of the TQF/DQF signals (*R*^*TQF*/*DQF*^) as a function of [Na]_i_ measured with shift reagent, in control hearts at baseline [Na]_i_ and in the four groups displaying elevated [Na]_i_ after treatment with 50 μM ouabain (n = 6); K^+^-free (n = 3), K^+^-free/Ca^2 +^-free (n = 3) and K^+^-free/Ca^2 +^-free/Mg^2 +^-free (n = 3). The mean *R*^*TQF*/*DQF*^ was 3.0 ± 0.3 for control hearts, 4.4 ± 0.7 for 50 μM ouabain treatment, 5.2 ± 1.1 for K^+^-free hearts, 7.7 ± 1.1 for K^+^-free/Ca^2 +^-free and 6.3 ± 0.4 for K^+^-free/Ca^2 +^-free/Mg^2 +^-free with a measured [Na]_i_ = 15.7 ± 1.6 mM; 25.5 ± 5.8 mM; 40.0 ± 5.7 mM, 57.9 ± 2.4 mM and 54.9 ± 6.5 mM, respectively. *R*^*TQF*/*DQF*^ showed a good correlation with [Na]_i_. The intercept on the y-axis demonstrates a non-zero *R*^*TQF*/*DQF*^ at [Na]_i_ = 0 mM confirming that there is a non-zero contribution from [Na]_e_ to the TQF signal. From this line of fit, intracellular Na can be estimated from a measured *R*^*TQF*/*DQF*^ according to the equation: [Na]_i_ = (*R*^*TQF*/*DQF*^ − 1.42) / 0.10. This suggests that the extracellular contribution to the TQF signal is ~ 47%, therefore slightly greater than the 32% contribution estimated earlier in the presence of shift reagent, but lower than previously reported values for the rat heart of ~ 64% [13].

**Fig. 6:**
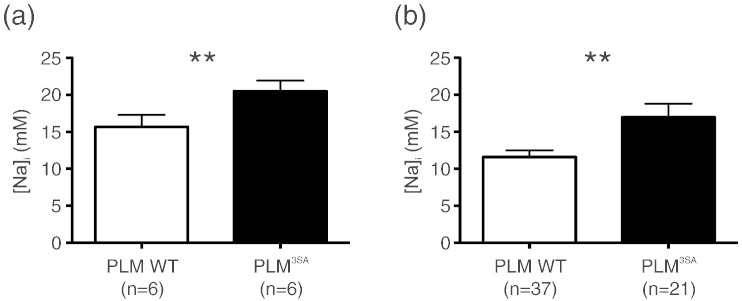
Plot of measured [Na]_i_ in the WT mouse and PLM^3SA^ transgenic mouse using (a) the shift reagent technique and (b) the derived values of [Na]_i_ using the TQF/DQF ratio. Significant differences between the two groups are shown **p < 0.05.

Fig. 6 shows intracellular [Na]_i_ measured in a cohort of wild type mice and in the PLM^3SA^ transgenic model. Fig. 6(a) shows [Na]_i_ measured with the shift reagent technique for the two groups, with a measured [Na]_i_ = 15.7 ± 1.6 mM (n = 6) in the WT and [Na]_i_ = 20.5 ± 1.4 mM (n = 6) in PLM^3SA^ hearts (p = 0.04). For the same cohorts and employing the calibration curve in Fig. 5, the TQF/DQF ratio was *R*^*TQF*/*DQF*^ = 2.79 ± 0.19 in the WT group with a derived value of [Na]_i_ = 13.7 ± 1.9 mM (n = 6) and *R*^*TQF*/*DQF*^ = 2.95 ± 0.18 in the PLM^3SA^ group with a derived [Na]_i_ = 15.3 ± 1.8 mM (n = 6) (p = 0.54). Despite the poorer signal-to-noise ratio in the multiple quantum filtered experiments the standard errors were only marginally greater for the TQF/DQF ratio compared to shift reagent, reflecting similar heart-to-heart variability of the two techniques. However the TQF/DQF ratio did not measure a significant difference in [Na]_i_ using a small n number. Using a larger pooled sample numbers the TQF/DQF ratio was *R*^*TQF*/*DQF*^ = 2.59 ± 0.09 in the WT group with a derived value of [Na]_i_ = 11.6 ± 0.9 mM (n = 37) and *R*^*TQF*/*DQF*^ = 3.12 ± 0.18 in the PLM 3SA group with a derived [Na]_i_ = 17.0 ± 1.8 mM (n = 21) (p = 0.024), Fig. 6(b). The TQF/DQF ratio required a larger pooled number of hearts to show a significant difference between the groups. A modest but significant elevation of baseline [Na]_i_ was measured in the PLM^3SA^ mouse compared to WT using both techniques.

## 4 Discussion

We have developed an MR-compatible Langendorff constant-pressure/constant flow perfusion system to non-invasively probe mouse myocardial [Na]_i_ at physiological heart rates and temperatures. Multiple quantum filtering techniques (DQF and TQF) were used to monitor [Na]_i_ during baseline physiological perfusion and during relative changes in [Na]_i_ initiated by inhibition of the Na/K ATPase activity by 50 μM ouabain and modified KH buffers (K^+^-free, K^+^-free/Ca^2 +^-free and K^+^-free/Ca^2 +^-free/Mg^2 +^-free KHB). Using the commonly used shift reagent, Tm(DOTP)^5 −^ to distinguish the relative contributions to the MQF signal from intra [Na]_i_ and extracellular [Na]_e_, we were able to quantify [Na]_i_ and thereby compare these to the measurements obtained using the MQF experiments. In the presence of shift reagent we found that the proportion of the total TQF signal arising from the extracellular [Na]_e_ pool was approximately 32%, indicating that the signal mainly represents [Na]_i_. We observed that the DQF signal was independent of elevations in [Na]_i_, consistent with this signal arising from the extracellular [Na]_e_ pool. In both TQF and DQF multiple quantum filtered experiments the bulk isotropic Na signal is filtered out of the spectrum leaving only the Na pools subject to anisotropic interactions; the TQF arising due to biexponential relaxation and macromolecular Na binding in the intracellular matrix whilst the rank 2 DQF signal arises due to a residual quadrupolar coupling due to association with ordered structures such as tissue fibre orientation. The relative amplitudes of the TQF and DQF signals, with the former being greater than the latter, represent the different anisotropic environments found in these different compartments. We found a good correlation between the TQF/DQF ratio R^TQF/DQF^ and [Na]_i_ and propose, therefore, that this ratio can be used as a semi-quantitative measure of [Na]_i_ in the mouse heart. Using the DQF signal as an internal extracellular reference on a heart-by-heart basis renders the TQF/DQF ratio independent of heart weight (data not shown), does not require an external [Na]_ref_ standard for calibration and enables a direct measure of [Na]_i_ without resorting to the use of shift reagents.

Absolute quantification of [Na]_i_ is challenging, particularly in the perfused mouse heart under physiological perfusion conditions. For the calculation of intracellular volume we assume a value of 2.5 ml/g dry weight of tissue which is adequate for the present control group of hearts [20]. This value is also in close agreement with that of 0.44 ml/g wet weight estimated by Jelicks and Gupta [24]. However, exact measurement of intracellular volume would be required if these techniques are to be applied under conditions where this might change. It must also be noted that a large proportion of the intracellular pool may not be visible due to the quadrupolar nature of the intracellular ^23^Na^+^ bound to macromolecules. The NMR invisibility of a fraction of the intracellular Na has been reported by some [25] whilst others have reported that intracellular Na is 100% visible [24].

Whilst the extent of ^23^Na visibility may bias the absolute quantification of [Na]_i_ using shift reagent [25], multiple quantum filtered ^23^Na will be useful to examine relative changes in [Na]_i_ applied to perfused mouse hearts. Both shift reagent and the TQF/DQF ratio were able to distinguish similar baseline differences in [Na]_i_ in the two groups of hearts. The PLM^3SA^ mouse displayed significantly elevated [Na]_i_ as measured both by shift reagent and the TQF/DQF ratio. Despite the poorer signal-to-noise ratio of the TQF and DQF NMR measurements compared to shift reagent (Fig. 3) the standard errors in the measurements of [Na]_i_ were similar in magnitude for the two techniques and only slightly larger for the TQF/DQF ratio, reflecting similar heart-to-heart variability. However there is a discrepancy in the absolute magnitude of the measured [Na]_i_ using the two techniques and a larger pooled sample size was required to reach significance between the two groups employing the TQF/DQF ratio. This may reflect the different measurement techniques and possible errors introduced in the derivation of the calibration curve as well as the different perfusion conditions, the groups measured with shift reagent being non-contracting due to the Ca chelation effect of the shift reagent whilst the TQF/DQF measurements are performed under more physiological conditions as reflected by the stable ex vivo cardiac function in Table 1. In future experiments, if absolute quantification of [Na]_i_ is not required but relative changes with respect to baseline then the % change in TQF signal alone could also be used, providing there is no change in the DQF signal, and would also offer a viable and more reproducible alternative to the TQF/DQF ratio proposed here. Whilst the TQF/DQF measurements required a large n number to distinguish baseline differences in [Na]_i_, the methods developed and validated in our study do not require the use of shift reagent, thus are performed under more physiological conditions, making our study readily applicable to the study of [Na]_i_ regulation in a wealth of transgenic models and muscle types with chronically altered [Na]_i_.

## Disclosures

None.

## Figures and Tables

**Table 1:** Cardiac function acquired at 5 min intervals during the 20 min stability period.

Functional parameters	Time (min)				
(n = 7)	0	5	10	15	20
Systolic pressure (mm Hg)	109 ± 12	113 ± 11	103 ± 17	103 ± 19	106 ± 19
EDP (mm Hg)	11 ± 1	9 ± 2	11 ± 2	10 ± 2	12 ± 2
LVDP (mm Hg)	98 ± 12	104 ± 11	92 ± 16	93 ± 18	93 ± 19
Heart rate (bpm)	382 ± 32	526 ± 54	453 ± 67	449 ± 79	464 ± 96
Coronary flow (ml/min)	2.2 ± 0.1	2.2 ± 0.2	2.4 ± 0.2	2.2 ± 0.2	2.6 ± 0.2

EDP — end diastolic pressure, LVDP — left ventricular developed pressure.
